# Supplementary material for: Integrins Increase Sarcoplasmic Reticulum Activity for Excitation—Contraction Coupling in Human Stem Cell-Derived Cardiomyocytes
Source: Int J Mol Sci. 2022 Sep 19;23(18):10940. doi: 10.3390/ijms231810940 (PMC9504605; doi:10.3390/ijms231810940)
Supplement: Supplementary file 1 [file ijms-23-10940-s001.zip › ijms-1832647-supplementary/Supplementary information.pdf]

Supplementary Materials for

Integrins increase sarcoplasmic reticulum activity for excitation-contraction coupling in human stem cell-derived cardiomyocytes

Brian X. Wang, MBBS, PhD, Christopher Kane, MBBS, PhD, Laura Nicastro, BSc, MRes, Oisín King, MSc, Worrapong Kit-Anan, PhD, Barrett Downing, MSc, Graziano Deidda, PhD, Liam Couch, MBBS, PhD, Christian Pinali, PhD, Anna Mitraki, PhD, Kenneth T. MacLeod, PhD and Cesare M. Terracciano, MD, PhD*

This file includes:

Online Figure S1 (Figure S1)
Online Figure S2 (Figure S2)
Online Figure S3 (Figure S3)

Legends for Movies S1 to S5

Other supplementary materials for this manuscript include the following:

Movies S1 to S5

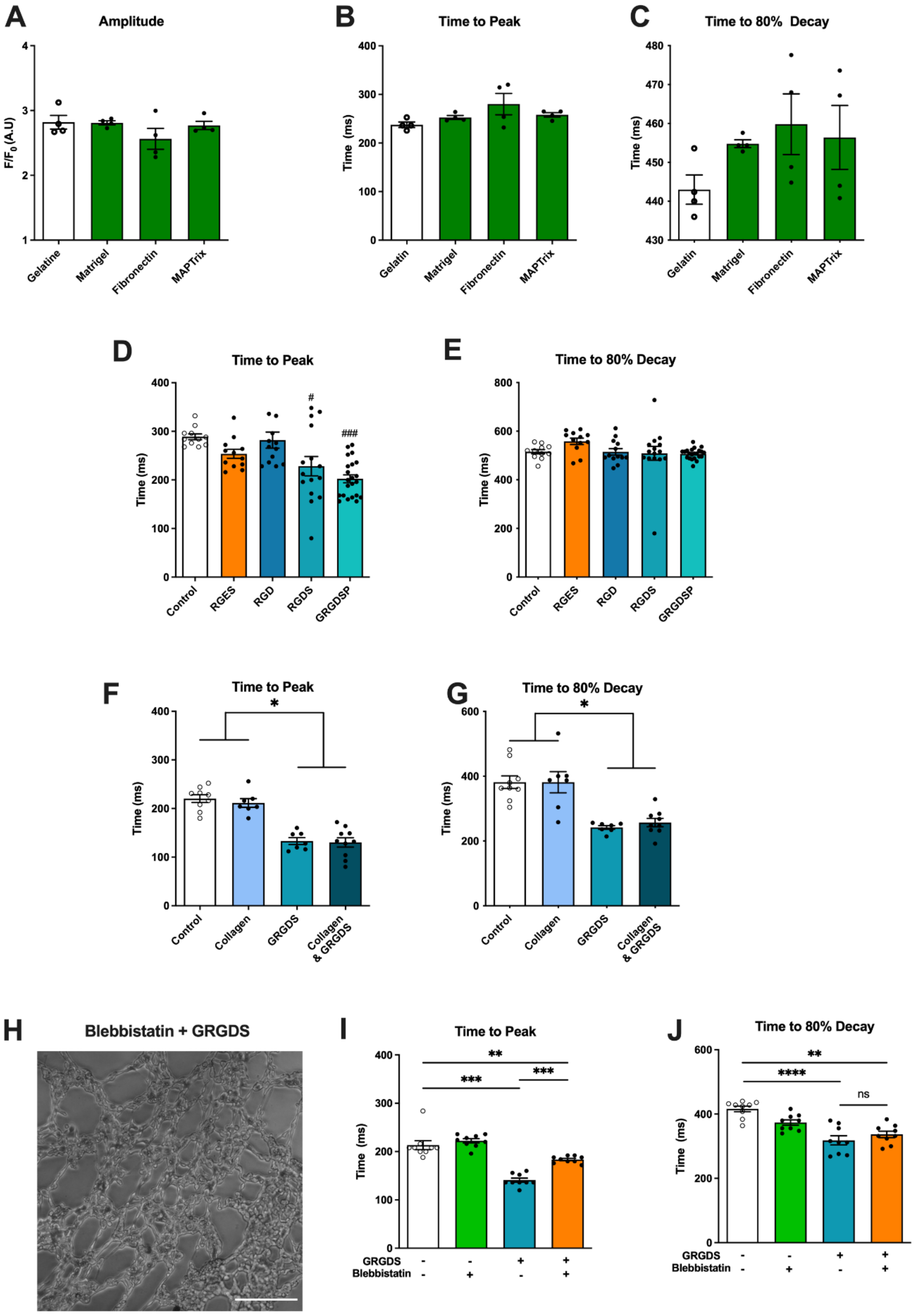

Supplementary Figure S1 Effect of extracellular matrix substrates and proteins on Ca^{2+}

handling properties. *Cardiomyocytes were cultured on dishes with solutions containing extracellular matrix proteins for 24 hours. Parameters measured were* Ca^{2+} *transient amplitude (**A**), time to peak (**B**) and time to decay (**C**). Data are represented as mean ± SEM. n = 4 recordings from 4 batches. Cardiomyocytes incubated with soluble RGD (Arg-Gly-Asp), RGDS (Arg-Gly-Asp-Ser), GRGDSP (Gly-Arg-Gly-Asp-Ser-Lys), GRGDS (Gly-Arg-Gly-Asp-Ser) or negative control RGES (Arg-Gly-Glu-Ser) for 24 hours before assessment of* Ca^{2+} *transients. Parameters measured were* Ca^{2+} *transient (**D**) time to peak and (**E**) time to 80% decay. # P = 0.0263, ### P = 0.0002 (n = 10-12 recordings with averaged signal from within the camera field of view from 4 batches). Cardiomyocytes treated with soluble GRGDS (Glycine-Arginine-Glycine-Aspartic Acid-Serine) (2 mM) and/or collagen gel (3 mg/mL) for 24 h.* Ca^{2+} *transient parameters after 24 h GRGDS treatment (2 mM) under 1 Hz field-stimulation. Cardiomyocytes were covered with a type I collagen gel, GRGDS solution or GRGDS-collagen solution (See Movie S3).* Ca^{2+} *transient (**F**) time to peak and (**G**) time to decay (n = 9 control, n = 7 Collagen, n = 7 GRGDS, n = 10 Collagen & GRGDS images that had signal from within the field of view averaged, from 3-4 batches). Modulation of GRGDS-induced* Ca^{2+} *handling changes by blebbistatin (**H-J**). (**H**) Pre-treatment with blebbistatin before GRGDS caused the cardiomyocyte monolayer to form a stellate pattern after 24 h.* Scale = 100 μm. *Cardiomyocytes were pre-treated with 5 μM blebbistatin for 1 h before GRGDS treatment and compared to control. Parameters were (**I**) Time to peak and (**J**) Time to 80% decay (n = 9 control, n = 9 Blebbistatin, n = 9 GRGDS, n = 9 GRGDS + Blebbistatin images, 3 batches).* * = $p < 0.05$, ** = $p < 0.01$, *** = $p < 0.001$, **** = $p < 0.0001$.

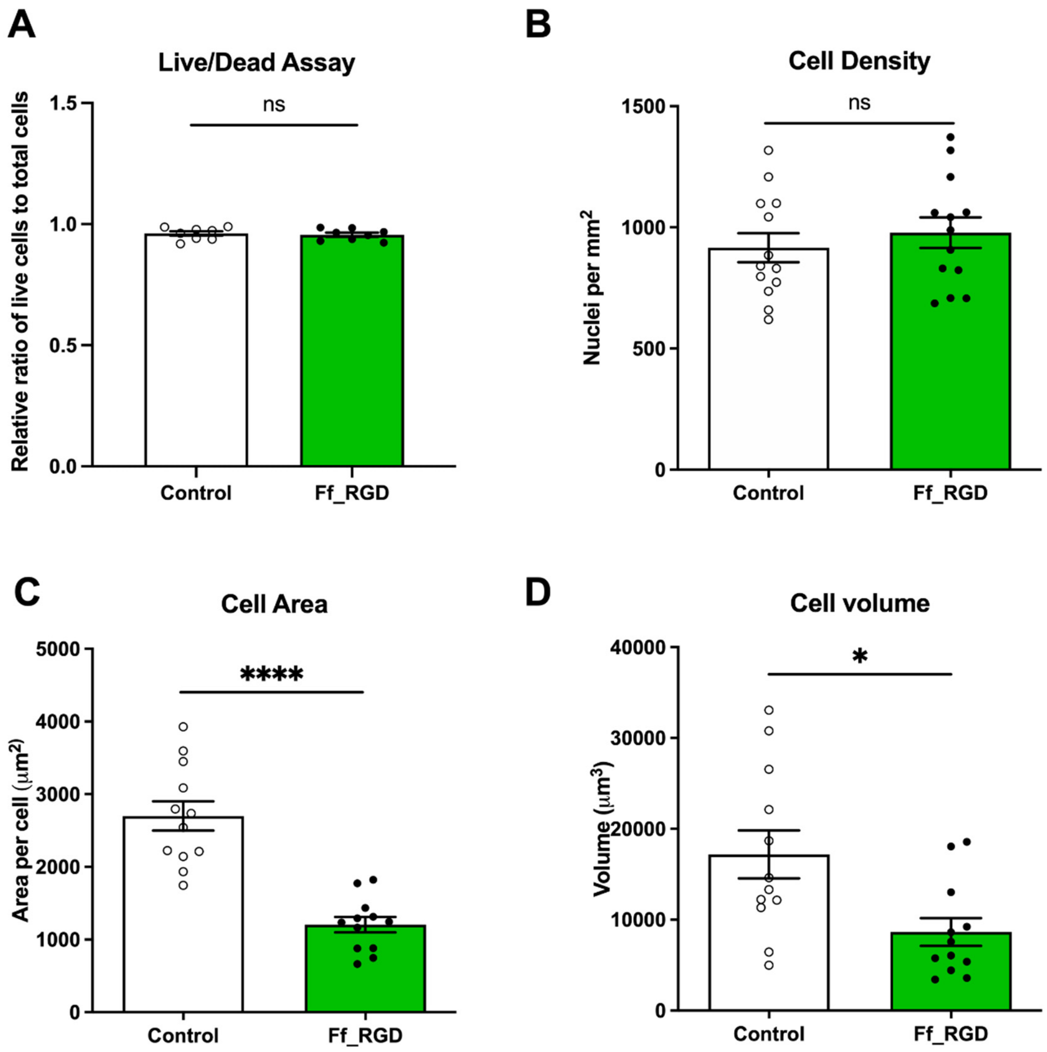

Supplementary Figure S2: Effect of ff_RGD on cell viability and morphology. *Cardiomyocytes were treated with ff_RGD for 24 hours and compared to control. (**A**) Relative ratio of live cells as a proportion of total cells, measured as the number of live and dead cells within the camera field of view (ns P = 0.691) (n = 8 control, n = 8 ff_RGD images from 4 batches). Confocal images were used to investigate (**B**) Cell density (ns P = 0.484) (n = 13 control, n = 13 ff_RGD images from 4 batches), (**C**) cell area (**** P < 0.0001) and (**D**) cell volume (* P = 0.0103) illustrated as means ± SEM (n = 12 control, n = 12 ff_RGD images from 3 batches).*

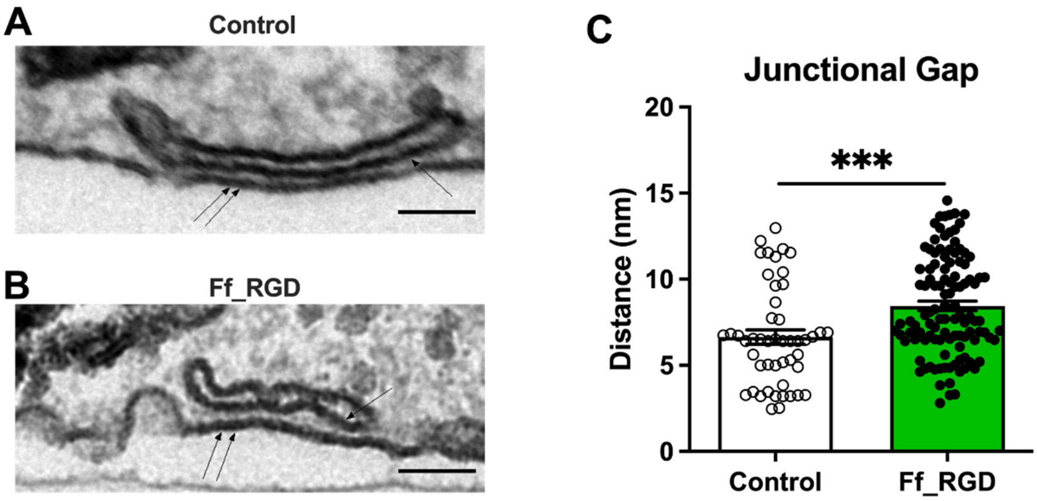

Supplementary Figure S3: Integrin ligand ff_RGD induced changes in the Ca^{2+} cycling ultrastructure closer to the adult phenotype. *Electron microscopy of (**A**) Control and (**B**) Ff_RGD-treated cardiomyocytes. Average distance between sarcolemma (double arrow) and junctional SR (single arrow) used to calculate (**C**) Junctional gap (*** P = 0.0004) illustrated as means ± SEM (n = 49 control, n = 111 ff_RGD cells from 3 batches). Scale bar = 100 nm.*

Movie legends

Movie S1 (separate file). Representative recording of a representative hiPSC-CM monolayer under control conditions. Scale = 100 μm.

Movie S2 (separate file). Representative recording of a representative hiPSC-CM monolayer after 24 h treatment with soluble GRGDS (2 mM). Scale = 1 mm.

Movie S3 (separate file). Representative recording of a representative hiPSC-CM monolayer after 24 h treatment with collagen-GRGDS (2 mM). Scale = 1 mm.

Movie S4 (separate file). Representative recording of a representative hiPSC-CM monolayer after treatment with ff_RGD (2 mM). Scale = 100 μm.

Movie S5 (separate file). Micropipette-based application of caffeine to a representative hiPSC-CM monolayer loaded with the Ca^{2+}-sensitive fluorescent dye fluo-4AM. Three field-stimulated twitch Ca^{2+} transients followed by a caffeine-induced Ca^{2+} transient. Application of caffeine in this fashion elicited a robust Ca^{2+} transient localised to the area of flow. Scale = 100 μm.
